# The Morality of Care: Female Family Caregivers’ Motivations for Providing Care to Older Migrants

**DOI:** 10.1177/10497323241280239

**Published:** 2024-11-05

**Authors:** Sunita Shrestha, Sanjana Arora, Alistair Hunter, Jonas Debesay

**Affiliations:** 1 Faculty of Health Sciences, Department of Nursing and Health Promotion, 60499 Oslo Metropolitan University, Oslo, Norway; 2 Centre for Intercultural Communication, 87446 VID Specialized University, Stavanger, Norway; 3 School of Social and Environmental Sustainability, 3526 University of Glasgow, Dumfries, UK

**Keywords:** aging, family caregiver, migrant, migration, morality, religion

## Abstract

Finding suitable long-term care arrangements for older migrants in Europe, including Norway, has been a major concern for healthcare policymakers in the last decade. However, many older people with migrant backgrounds, and to a certain extent their descendants, often prefer that care arrangements are managed within the family. Although caring for family members may be personally satisfying, it can also be a source of distress. This study explores the motivations of care among female family caregivers of older Pakistani migrants within the Norwegian Ahmadiyya community. Our data are derived from a qualitative study including individual and group interviews with 19 female family caregivers. The study participants were aged 25–62 and resided in Norway. The interviews were conducted in Urdu and English and later transcribed verbatim in English. Our findings resulted in four main themes regarding motivations for caregiving: (1) “Who else, if not the family?”: care perceived as a family obligation; (2) The divine duty of caregiving; (3) Women are better at caregiving; and (4) “What will people say?”: fear of judgments. The intersection of culture, religion, gender, and migration shaped caregiving as a moral practice, and those providing care were considered individuals with high moral identity. The moral identity assigned to the role of family caregivers appears to exacerbate rather than alleviate the care burden on women of migrant origin. Understanding the motivations for caregiving can shed light on ways in which better support can be provided to ethnic minority families with aging members.

## Introduction

There has been a significant increase in the population of migrants in Europe from non-European countries since the late 2000s (WHO, 2018). Though many are young adults, the number of older migrants is increasing in Norway (Syse & Tønnessen, 2022). Compared to the general population, non-European migrants are potentially more disadvantaged due to lower socioeconomic status, language barriers, and limited access to appropriate health services (Debesay et al., 2023; WHO, 2018). Additionally, fewer older people with migrant backgrounds use nursing homes, and many prefer to be cared for by their family members, thus increasing the family caregivers’ care responsibilities (Arora et al., 2020; Nielsen et al., 2021).

### Women as Caregivers of Older Adults

Caregiving ranges from assistance with daily activities, and emotional support, to helping to navigate complex healthcare and social service systems (Schulz et al., 2020; Zarzycki et al., 2022). Though family caregiving is associated with personal growth, self-efficacy, satisfaction, a sense of gratification, and a closer relationship with care receivers (Zarzycki et al., 2023), many studies also showed that caregivers have poorer health outcomes than non-caregivers, ranging from physical challenges to chronic stress, anxiety, social isolation, and limited social circle (Schulz et al., 2020; Stenberg & Hjelm, 2023). Gender disparities in the distribution of caregiving duties often result in a higher burden of care among women in general (Zygouri et al., 2021). Such disparities are even higher among people with migrant background (Shrestha et al., 2023; Tavernier & Draulans, 2018) where spouses, daughters, and daughters-in-law shoulder more care responsibilities. In particular, women have a higher emotional burden, such as feelings of distress and hopelessness, and a sense of loss of the relationship, as women focus more on emotion and men tend to be more task-oriented in caregiving (Calasanti, 2010; Zygouri et al., 2021).

### Family Caregiving Among Migrants

Caregivers with migrant backgrounds tend to express caregiving as part of cultural norms, filial obligations, reciprocity, and moral obligations (Shrestha et al., 2023). Unwillingness or failure to follow such norms leads to guilt, shame, and social exclusion among caregivers (Tavernier & Draulans, 2018). Thus, many caregivers with migrant backgrounds may downplay their own needs while adhering to caregiving norms (Tavernier & Draulans, 2018) and still many adult children feel responsible for caring for their older migrant parents, although intergenerational caregiving dynamics are shifting, from sole toward shared care responsibilities (Shrestha et al., 2023).

Norway has a strong welfare state that is mandated to provide health and social care for older adults (Skinner & Sogstad, 2022). With the increasing aging population, there is greater pressure on the welfare state to manage their health and social care. Hence, there has been a strong policy emphasis on increasing voluntary contributions and better integration of informal care with public care services in recent years (Skinner & Sogstad, 2022). While formal elder care is the norm among the majority of the Norwegian population (SSB, 2020), it is estimated that a large share of the older population with migrant backgrounds, particularly among the Pakistani population, is still highly dependent on family members for care in their old age (Næss & Vabø, 2014; Sagbakken et al., 2018).

The Pakistani population in Norway constitutes one of the largest and longest-residing groups among non-European migrants, and older Pakistanis are one of the largest groups of older migrants (Arora et al., 2020; Ingebretsen et al., 2015). As people age, they require more care due to multimorbidity (WHO, 2018). These increased care needs result in more family members taking the role of caregiver (Arora et al., 2020). However, studies on informal caregiving with a primary focus on the family caregivers of older migrants are limited in Norway. Overall, there is limited research on how family caregivers of older migrants conceptualize and negotiate ideas of “good care” (Horn, 2023).

### Ahmadiyya Muslims in Norway

The first Pakistani migrants arrived in Norway in the late 1960s and early 1970s as labor migrants, followed by family reunification (Brochmann & Kjeldstadli, 2008). Ahmadis, a persecuted religious minority from mainstream Muslims from Pakistan, migrated as refugees to Norway in the 1970s, followed by family reunification. The first Ahmadi missionary arrived in 1958 (Jacobsen et al., 2015), and there are about 1200 Ahmadi Muslims in Norway, mostly of Pakistani origin, residing in Eastern Norway (Høydal et al., 2023).

The Ahmadis are a religious minority in Pakistan, where the majority are Sunni Muslim. In Norway, they are a minority within the larger Pakistani Muslim minority (Jacobsen et al., 2015). The Ahmadis in Norway are thus a minority within the minority, as they are a religious minority within a larger ethnic minority (Pakistani Muslim). This double minority status leads to their misrecognition by state and societal actors who often overlook intra-ethnic diversity (Galal et al., 2016). Furthermore, mainstream Muslims often avoid incorporating Ahmadi voices in their representation of Muslims, viewing their beliefs as heterodox. Consequently, Ahmadis are denied membership in the Islamic council of Norway, an umbrella organization consisting of more than 40 member organizations (Leirvik, 2014; Naserah, 2022). They continue to face discrimination and hateful expression not only in Pakistan but also in Norway (Færseth, 2021; Naserah, 2022) and other countries with significant Muslim populations like the United States, the United Kingdom, Switzerland, and Germany (Ahmed-Ghosh, 2004, 2006; Kelso, 2023; Langewiesche, 2020; Yaqin et al., 2018). Participants in this study mentioned staying informed and vocal about these phenomena through social media and communication with fellow Ahmadis in Pakistan and other diasporas.

The Ahmadis remain a “double minority,” not simply due to their lower numbers but in relation to their lower status in the eyes of the mainstream Muslim community (Galal et al., 2016). Consequently, their persecution narratives featuring heavily in Ahmadi discourses have shaped their unity and also their representation in the Western world as “good Muslims” (Yaqin et al., 2018). In Europe, they are seen (by majority society) as better integrated due to their positive visibility through strong interfaith relationships, loyalty to the host country, education for women, advocacy for peace, and self-representation as true Muslims (Appleyard, 2015; Islam, 2019; Kelso, 2023).

### Theoretical Frameworks: Ethics of Care and Intersectionality

To understand caregiving to older adults in the family as both a concept and a practical endeavor, we apply the theory of the ethics of care. It recognizes human relationships, interdependency, and mutual concerns (Gilligan, 1982; Noddings, 1984; Tronto, 1993). The ethics of care take a central stage in articulating morality and achieving care (Tronto, 2013). Tronto (1993) described care as having four phases, with a fifth added in 2013. These phases are analytically separate but interconnected, each linked to an ethical aspect of care. The first phase of care, “caring about,” involves recognizing another’s need, with attentiveness as the key moral element. The next phase, “caring for,” involves taking responsibility to meet the identified needs, with responsibility being the central moral quality. This responsibility is contextual (often gendered in a cultural context), action oriented, and relational in care ethics (Tronto, 1993, 2010). The third phase, “caregiving,” involves direct physical work and requires caregivers to be competent and resourceful. The fourth phase, “care receiving,” involves the response from the care recipient. The final phase, “care with,” emphasizes that care should align with democratic commitments to justice, equality, and freedom for all (Tronto, 2013). The ethics of care will help to understand how family members conceptualize their caregiving for older adults with migrant backgrounds.

Caregiving is often tied to femininity. Gilligan (1982) stressed women’s attitudes and inclination toward care as a construct of moral development, where women were more emotionally connected and driven by interdependent relationships and concern for others’ needs. Caregivers ignoring their own needs and concentrating more on achieving the needs of others is also often considered an ethical aspect of care (Noddings, 1984). However, such notions of care ethics closely tied to femininity and sacrifice have been criticized by gender scholars for not considering the power of social norms and institutions (Chang, 1996; Tronto, 1993; Weicht, 2015). For example, Chang (1996) emphasized that the morality of care is socially constructed, with power relations playing a central role. Those in power advocate rules and rationality, while those with less power espouse relationality and compassion (Chang, 1996), are too self-sacrificing, and are likely to be relatively powerless in society (Tronto, 1993). Thus, care is deeply implicated in the existing structures of power and inequality.

While care ethics primarily focus on the moral significance of caring relationships, empathy, and interconnectedness, they also recognize the presence of power dynamics within these relationships between caregivers and care receivers. Caregivers hold power over care recipients and advocate for the well-being, autonomy, and dignity of care recipients. Thus, there are dynamic and reciprocal relationships in caregiving where the needs, perspectives, and vulnerabilities of both the care receiver and the care recipient require attention (Tronto, 2013), which calls for an intersectional perspective on caring that considers the diverse experiences of caregivers in addition to gender and race. Feminist ethics of care are often critiqued for not acknowledging aspects of intersectionality with social positions of class and race (Hankivsky, 2014; Raghuram, 2019).

Intersectionality, rooted in black feminism and critical race theory, examines the interconnections of multiple social locations such as race, gender, religion, and class (Crenshaw, 1989, 1991). It sheds light on the lived experiences of overlooked groups, such as migrant group (Hankivsky et al., 2009), and focuses on multiple systems of power associated with intersecting identities (Brown et al., 2016; Cohen, 2021). Intersectionality broadens the perspective of the ethics of care by highlighting the multifaceted nature of an individual’s social locations and how these influence caregiving practices and ethical considerations (Raghuram, 2019; Ward, 2015). Recognizing the importance of caregivers’ social locations in lines of ethnicity, religion, gender, and migration, we adopt the intersectionality perspective to address their role in shaping caregiving ideals. As both care ethics and intersectionality share normative ideals of social justice, care ethics inspired by intersectionality investigate the influences of multiple social locations on power relations and resulting inequalities (Hankivsky, 2014; Hankivsky et al., 2009). Therefore, intersectionality is helpful in our study as an analytical lens to understand how the social locations of family caregivers, such as religion, gender, migrant status, and ethnicity, interact to shape the ideals of good care.

### Aim and Research Questions of the Study

A few studies in Norway have explored caregiving for migrants, predominantly concerning their access to formal healthcare services (Arora et al., 2020; Gulestø et al., 2023; Næss & Vabø, 2014; Sagbakken et al., 2018). Given migrants’ socioeconomic position in Norwegian society, combined with gendered caregiving norms, an intersectional approach (considering different social locations such as culture, religion, gender, and migration) is relevant when studying informal caregiving in migrant families. However, the influence of religious and gender perspectives on informal caregiving among migrants seems underexplored. To the best of our knowledge, no study has been conducted to explore caregiving motivations and experiences among this minority Muslim group. Thus, this study aims to explore the motivations and meanings of care among family caregivers of older Pakistani migrants within the Norwegian Ahmadiyya community. In doing so, we explore the significance of gender, religion, and migration in the continuation of family caregiving. This can shed light on ways in which better support can be provided to ethnic minority families with aging members, which is crucial for the well-being of both caregivers and care recipients. To address the aim of the study, the following research questions were formulated: What is the motivation and meaning of providing care to older family members in migrant families? How do family caregivers perceive their responsibilities of providing care to older family members in migrant families?

## Methods

An exploratory qualitative study using semi-structured interviews and observations was employed in this study which enabled us to collect in-depth information from female family caregivers. Our focus was on understanding the family caregivers’ subjective motivations and meanings of caregiving for older Pakistani migrants from the Ahmadiyya community in Norway. The explorative nature of the study sheds light on the significance of various social locations, such as gender, religion, and migration, in the continuation of family caregiving, a topic that has often remained underexplored.

### Recruitment of Study Participants

Several studies among the migrant population in Europe showed that women have a higher burden of care (Lillekroken et al., 2023; Shrestha et al., 2023; Stenberg & Hjelm, 2023), including among Pakistani migrants in Norway (Arora et al., 2020; Næss & Vabø, 2014). Therefore, the participants in our study were women from Ahmadiyya community aged 25–62 years old who provided unpaid care to older family members and had at least 6 months of experience as family caregivers.

Participants were recruited from Oslo municipality, which has the largest number of Ahmadiyya residents in Norway. For recruitment, we emailed the national president of Ahmadiyya Muslim Jamaat Norway, who put us in touch with the president and committee members of an Ahmadiyya women’s organization. A total of 19 female family caregivers were recruited to the study in August–November 2021 and September–November 2022. In 2021, nine participants were recruited through key informants, whereas one of the participants was recruited directly by the first author through social media (Facebook) after reading her news articles about the Ahmadiyya community’s issues. In 2022, four participants were recruited through the help of the key informants and five through snowball sampling.

We prioritized recruiting participants with different migration histories, ages, educational backgrounds, marital statuses, and generations to explain caregiving experiences based on their various social locations. More than half of the participants in our study were either first-generation migrants (nine participants) who arrived in Norway as adults through family reunification or 1.5 generation (Danico, 2004) Pakistani migrants (five participants) who migrated to Norway during childhood and had been living in Norway for at least 20 years. The remaining participants consisted of second-generation Norwegians born to immigrant parents (five participants) in Norway. Two-thirds of the participants were aged 40–60, and similarly, two-thirds were employed, either full-time or part-time. Their experience as primary caregivers for older family members ranged from 6 months to several years.

### The Interviews and Observations

The data were collected through 18 semi-structured individual interviews (16 individual interviews and two follow-up individual interviews) and two group interviews with two participants. In each group interview, the participants were either siblings or relatives (sister and sister-in-law), which enabled us to see the intra-generational/relational dynamics in conceptualizing care. The participants had varied relations to the older care receivers (daughters, daughters-in-law, sister, and granddaughter). We used a semi-structured interview guide to explore their motivation of family care and included questions such as: Could you tell me why children provide care for their aging parents/relatives? Could you explain why you chose/decided to be a caregiver to your mother/father/parents/in-laws/sister/grandparents? Why is it important for you to be a caregiver for your mother/father/parents/in-laws/sister/grandparents?

In group interviews, participants were more vocal regarding the care norms of their community and less about challenging caregiving experiences, unlike in individual interviews. Similarly, this pattern was observed when interviews were conducted at the Mosque. About half of the interviews (12) were conducted at a mosque, mostly during the first phase of participant recruitment. During the second phase, a deliberate choice was made to conduct interviews outside the Mosque, when possible, to minimize the potential influence of the Mosque location on the data collected. Therefore, the remaining interviews took place in public libraries (4) and via video calling on Microsoft Teams (4). All interviews were conducted by the first author in Urdu and English and were audio-recorded and later transcribed verbatim in English. The anonymity of the participants was maintained in all transcripts. Each interview lasted from 45 minutes to 3 hours (38 hours in total), which resulted in 375 pages of transcribed material.

The first author’s migrant background, South-Asian origin, Urdu-speaking skills, and familiarity with some aspects of the participants’ culture positioned her as an insider. At the same time, not being Pakistani or Ahmadiyya simultaneously positioned her as an outsider (Blix, 2015). Therefore, naturalistic observation was undertaken while participating in some of the events and exhibitions organized in the mosque. The observations helped facilitate informal conversations with the women and learn more about the Ahmadiyya community. The first author took notes and reflected on each event. However, data only from the two main events are included in this article. The first event was Jalsa Salana UK 2022, and the second event was the 100th anniversary program of the Ahmadiyya Women’s Organization. In both events, the worldwide head of the Ahmadiyya Muslim Community gave a speech to Ahmadiyya women regarding their roles and responsibilities in Islam in the present context.

### Data Analysis

We drew on Braun and Clarke’s reflexive thematic analysis (2022) to support our analysis. This included the following six activities: Our first step in the data analysis involved getting familiarized with each transcript. The first author familiarized herself with the data by repeatedly reading transcripts and listening to the audio recordings, noting immediate thoughts and ideas. Two reference transcripts were shared among the co-authors for familiarization. The second phase consisted of generating codes; first, all authors met to share/co-generate codes from the two reference transcripts. After that, the first author coded the remaining transcripts and organized the data around similar meanings. This coded data was further shared with and discussed among the co-authors.

In phase 3, the codes were organized into potential themes, where social locations such as ethnicity, gender, religion, and migration were kept in mind to explore their significance in maintaining and continuing informal caregiving for them. These themes were further discussed and reviewed by all authors in phase 4 and redefined in phase 5 to ensure their comprehensiveness to capture the essence of the data in relation to the research question. The final phase involved producing the report (Braun & Clarke, 2022). The data analysis process was iterative, reflexive, and combined inductive and deductive approaches. Table 1 presents the phases of thematic analysis and shows how we developed one of the study themes.

**Table 1.** Phases of Thematic Analysis and Generating Themes.

Phase of Thematic Analysis					
1. Familiarization with the data	2. Generating initial codes	3. Searching for themes	4. Reviewing themes	5. Defining and naming themes	6. Producing the report
-*We understand their heart and their feelings, and they have confidence that family members will take care the right way*. - *I am the oldest, and I still am the one who will take or make all the major sacrifices needed for the house because it just comes naturally*. -*You are born in that culture that everything will be automatic. Like when you get up in the morning, you know that you must go to the bathroom, brush your teeth, have breakfast, and go to work. Likewise, you know you must take care of your parents*.	-Family giving best care -Sacrificing for family is natural -It’s automatic to help family; no choice	-Taking care of their own -Family obligation -Filial piety	-Family obligation basis of familial relation	Who else if not family	Explaining “who else if not family” in findings

### Ethical Considerations

Ethical approval for the study was obtained from the appropriate ethical committee in Norway. All the participants were informed about the study and notified that they could withdraw from the study at any time and without giving any reason. Written consent was obtained in the case of face-to-face interviews, whereas for Microsoft Teams interviews, oral consent was received.

### Reflexivity

The authors have Asian, African, and European backgrounds. Two are men and two are women. All authors had prior experience doing research with migrants and migrant families and have academic backgrounds in nursing and social and health sciences. The authors with different academic competencies and research experiences, as well as different ethnic backgrounds and genders, added diverse perspectives while analyzing the data. Discussions among the authors helped clarify aspects of traditional care vis-à-vis patriarchal power dynamics in gendered relations.

### Rigor

In-depth interviews and active listening during those interviews further added to the quality of the data, where participants had enough time to share their experiences. Interviews were the best medium to voice opinions regarding the ideals of family caregiving. The interviewer’s background as an Asian Urdu-speaking migrant also appears to have instilled trust and facilitated a more candid dialogue with the study participants. Nineteen participants were involved in this study, encompassing a variety of social locations, including age, migration history, generation, education, occupation, and relationship to care receivers. Furthermore, the explorative nature of our study and the variation in caregivers’ characteristics allowed us to collect comprehensive information as well as generational similarities and differences regarding the motivations and experiences of informal caregivers of older migrants. Further, all authors contributed to constructing, defining, and redefining the themes through discussion until a consensus was reached, adding to the study’s credibility.

## Findings

Family caregivers provided physical, medical, emotional, and economic support to older adults. They were responsible for assisting with daily life activities, for example, household tasks such as cooking, cleaning, shopping, and personal hygiene. They also coordinated medical appointments and schedules, helped with transport to doctors’ appointments, and communicated between health personnel/health system/social benefits and their older family members. They entertained older adults by spending time with them and were also the source of emotional support that motivated them to do better health-wise.

Our analysis resulted in four themes: (1) “Who else, if not the family?”: care perceived as a family obligation; (2) The divine duty of caregiving; (3) Women are better at caregiving; and (4) “What people will say?”: fear of being judged.

### “Who Else, If Not the Family?”: Care Perceived as a Family Obligation

Most participants maintained that providing care to their parents was a natural part of life. “Who else, if not the family?” was the response often echoed during the interviews, which showed the high value of family solidarity, often referencing the family care norms of their or their parents’ country of origin, Pakistan. Stronger families were perceived to be those who stayed together when an older family member needed help, and such families had higher status in their community (*Jamaat*). Participant 6, who came to Norway after getting married and cared for her mother-in-law, believed that family is suitable for caring for older family members, as they are reliable and understand the emotional aspects of care. Thus, they are the ones who take care of family in the right way.

Caregiving was perceived as natural by several participants, both for those born in Norway and those who relocated after marriage. A participant who came to Norway as a child conveyed that caring for family comes naturally. She said:

You are born in that culture that everything will be automatic … like when you get up in the morning, you know that you must go to the bathroom, brush your teeth. This is automatic. Likewise, you know you must take care of your parents. That is our Pakistani culture. (Participant 2, age 40–50, migrated to Norway in childhood)

Thus, the participants often attributed culture (particularly parents’ culture) as shaping their outlook toward informal caregiving, as they have seen their mothers take care of their family and aging parents back in Pakistan as well as in Norway. Many visited Pakistan with their parents as children or saw their parents visit grandparents. Those with transnational marriages also visit their or their husbands’ parents. Furthermore, sacrifices from family members were both expected and naturalized in the context of informal caregiving. Another caregiver, participant 16, age 30–40 and born in Norway, said, “I am the oldest, and I still am the one who will make all the major sacrifices needed for the house because it just comes naturally. I do not think much about it.”

Many participants (who were daughters) who were either born in Norway or came here as children highlighted that the children, particularly daughters and sons, were responsible for caregiving rather than daughters-in-law. They were looking after their parents, even when their parents-in-law were also in Norway. However, this was not the case for participants who were daughters-in-law and came to Norway as adults. They still had the responsibility of taking care of their parents-in-law. Meanwhile, few participants who were born in Norway or migrated in childhood expressed it as unfair to daughters-in-law.

I am the daughter, and it is my responsibility, my brother’s responsibility, not sister-in-law’s responsibility. That is not right. The feelings we have for our parents, we cannot expect from others. If she wants to do it, she can do whatever, but I will not put the responsibility on her. (Participant 12, age 40–50, migrated to Norway in childhood)

Her words highlight that although children are obliged to take care of their parents, and as children they have no other options than taking care, others should not be forced into caregiving. Caregiving by children is also presented predominantly as a selfless act out of love, which is not always the case for daughters-in-law. In line with this, participant 1, aged 50–60, migrated to Norway in adulthood, who has experience of taking care of both her mother-in-law and mother, expressed caregiving to her mother-in-law out of respect and duty, whereas for her mother, it was out of respect and love.

Several caregivers also defined caregiving as reciprocal, where not paying back the care they had gotten from their parents would not be the right thing to do. Such feelings were more pronounced among those participants who had a socially and economically challenging childhood and experienced their first-generation parents’ struggles with limited language skills and social circles, not to mention precarious working conditions. Several participants had older relatives with debilitating health, which made them feel obliged to look after them in return. Not providing care was viewed as ruining the old age of their parents. The feeling of being morally obliged to look after the vulnerable was consistent among many caregivers across generations. The ones who were recently joined by their elderly parents from Pakistan expressed being even more morally obliged as their parents were new to Norway, and unfamiliar with the Norwegian context, which makes them even more vulnerable.

### The Divine Duty of Caregiving

Many participants perceived caregiving as a moral duty and believed that religion had an important role in influencing ideas of morality. Some participants recalled regularly visiting the Mosque since childhood and learning these care ideals with other children of similar age. They believed that older adults have a high value in Islam and have the right to be cared for with utmost respect by their children. Participant 6, age 50–60 and migrated to Norway in adulthood, perceived providing care as a way to maintain a connection with Allah as a moral being by obeying her duties. She added, “Allah provides everyone food for his part, and you have to do your part of the duty.”

The moral aspect of caregiving makes it less likely for some children to abandon their care responsibilities for their parents. Participant 4, age 60–70 and migrated to Norway in adulthood, stated, “For us [as compared to Norwegians] we do not have background [referring to Pakistani care norms] to keep elderly at nursing home. If they get sick also, they stay at home and become dear to God [die].” Thus, it was considered a disgrace and dishonor for older adults as well as a matter of shame for many participants, such as Muslims, to hand over the responsibilities of older relatives to others. Maintaining good care for their older family members was essential for some caregivers, as they perceived that they were answerable to God. Participant 7, age 60–70, migrated to Norway in adulthood, specified, “We even cannot talk in a loud voice. It is forbidden in Islam to talk at a higher volume or to get angry with our parents. It is said in the Quran.” Hence, characteristics such as tolerance, courage, and sacrifice were repeatedly cited by participants as good caregivers’ qualities.

Participants also relied on religion to make sense of the challenges associated with caregiving. Participant 11, age 50–60, migrated to Norway in adulthood, expressed the importance of tolerance and courage during caregiving, as her mother had shown while caring for both her parents and parents-in-law in Pakistan and believed that God does not burden anyone beyond their capacity. Participant 16, age 30–40, born in Norway, attributed her good grades at university to her being a caregiver and quoted, “My mom said that it was all the prayers and the fact that you sacrificed so much, and God saw it, and that is why you got it. I think I really did gain some points by being her caretaker.” Hence, the sacrifices made by caregivers by prioritizing parents’ care were perceived as a way of earning blessings, which, in turn, would lead to something good in the future (Good Karma).

### Women Are Better Caregivers

Several participants spoke about their significantly higher share of providing care compared to men in the family. They rationalized the care division as being due to men and women being perceived as having different hierarchies and different responsibilities. Participant 8, age 40–50, migrated to Norway in adulthood, mentioned, “Same as we do not have equal fingers. Every person’s level is different. Men have their position, and women have their own. Allah has made us that way.” She further added, “I think at home Allah has made woman the ‘queen of the house’ to look after household work.” The public–private space analogy and corresponding gender tasks were highlighted by some other participants as well, including those born in Norway. Participant 15, age 20–30, born in Norway, mentioned having Pakistani culture at home where, unlike her father and male siblings, she has no economic responsibilities but, like her mother, she is responsible for care and emotional contribution. Religion has often been utilized to naturalize such gender differences.

Many participants also described themselves as different and better caregivers than their partners or male siblings. They perceived women as better caregivers because they multitask and can endure challenges. Characteristics like naturally caring, tolerant, patient, sacrificing, selfless, prioritizing others, and caring from the heart were the expressions many caregivers used to describe themselves. Many participants also perceived themselves as those who valued relationships and were more emotionally attached to their family members “naturally” than their male siblings. Religion was also cited to explain women’s better caregiving skills and capacity.

I think Allah made women like this in every culture and in every religion. Women are more delicate [than men], so we think about everything delicately and with heart. So, when it comes to parents’ health or taking care of them, they [daughters] will do it right away. (Participant 2, age 40–50, migrated to Norway in childhood)

Furthermore, she shared an incident when her mother became severely sick due to an infectious disease. While her brothers were discussing dividing duties among siblings, she had already reached her parents’ house to help them. Similarly, participant 15, age 20–30, born in Norway, felt responsible for leaving her job to care for her mother, even though she has two older brothers. She shared that she is willing to do a less demanding job or reduce her employment responsibilities in the future if she takes care of her mother more. Thus, the participants perceived caregiving as intrinsic and natural to women, which made them feel more responsible for taking up care initiatives and tasks, irrespective of their own personal circumstances or professional ambitions. Some participants spoke about modesty/Purdah considerations in Islam, which limited their capacity to care for older male members of the family. Despite this, they perceived themselves as responsible for all of their care apart from hygiene-related issues.

Acceptance that household and family are women’s spaces was described by some participants as a prerequisite for avoiding conflicts and ensuring a peaceful environment at home. One of the participants who came to Norway as a child and was employed mentioned the potential for backlash from husbands and male relatives if women would question the inequality in private spaces. Participant 2, age 40–50, migrated to Norway in childhood, added, “It should not be unfair, but why do we need more than that? Then, it is like shooting yourself in the foot.” Similarly, participant 1 emphasized taking the role of caregiver for her mother-in-law to maintain a good relationship at home. She stated:

Since I am a daughter-in-law, it is my responsibility. If they do not, it clashes, and people fight in the house. If people understand this and want to be with their husbands, they must also fulfill their duties. (Participant 1, age 50–60, migrated to Norway in adulthood)

Caregiving as a woman was also perceived as a way to set an ideal example to teach children, particularly girls, the importance of family caregiving. Participant 7, age 60–70, migrated to Norway in adulthood, said, “we try our best to care for them [parents]. My daughter has been observing how I take care of my mother. So, she has this thing inside her now that she never gets irritated when she stays with my mother.”

Thus, conducting oneself as an ideal caregiver, signifying the one with high moral values, was considered vital for maintaining the generational continuity of informal care. Such perceptions were also echoed in events at the women’s organization by community and religious leaders.

### “What Will People Say?”: Fear of Being Judged

The participants spoke about the high importance that the Ahmadiyya community ascribes to family care. Participant 12, age 40–50, migrated to Norway in childhood, mentioned the social pressure to fit within her community with higher values of Pakistani culture and recalled her childhood memories of her parents’ constant effort to avoid being influenced by Norwegian culture. As a result, she was sent to Pakistan for a few years to stay and to learn the homeland’s values, culture, practices, and language, while her brother stayed in Norway. This was a similar story for a few other participants, too.

Some other participants described their community as close-knit, where “everyone knows everyone.” Thus, many participants felt that other community members tended to check on how well they were taking care of one’s parents. This surveillance intensified the need to adhere to standard norms within the community, with care ideals from Pakistan serving as a reference point. Some were also concerned about negative reactions from the community if they did not adhere to the care norms. Participants were highly concerned about not being able to fulfill their care responsibilities or either seeking or choosing alternatives to family care and expressed, “What will people say if we do so?” One of the participants who came to Norway as a child further stated about the gossip that could go around in the community that could affect the respect and prestige people or family have. Although people could seek alternative care solutions, there was also a fear that there would be consequences if people deviated from the social norms rooted in culture and religion.

They are too scared to lose respect in society. People will say, “Oh, this is happening in his family. We thought everything was perfect.” So, you know there was a film “what will people say” [referring to gossip and interference in other families’ affairs]. That kind of pressure we get from society is there. (Participant 2, age 40–50, migrated to Norway in childhood)

Thus, community approval was viewed as a source of respect and status that was, in turn, connected with informal caregiving. For all participants, it was important to uphold their identity as good daughters/daughters-in-law and Muslims through being good caregivers. Being vocal about the challenges of caregiving was seen in conflict with such identities and often resulted in getting judgmental remarks from elder members of the community. In addition to fellow Ahmadis’ perceptions of them, many participants were also concerned about how they, as Ahmadiyya Muslim women, were viewed by other Pakistanis (mainstream Muslims community) and Norwegians. For example, one participant stated:

It is a stigma because you are supposed to keep everything under the carpet and not talk about it, and if you do, they think about you as not a good person at all. You are not a good Muslim or a good Pakistani, and you are also not a good daughter if you do that. (Participant 5, age 50–60, migrated to Norway in childhood)

Similarly, some women spoke about how being caregivers for their older family members made ethnic Norwegians perceive them positively, contrary to the general negative preconceptions about Muslims, and particularly Muslim women due to them wearing hijabs and thereby appearing oppressed. Most participants usually referred their caregiving to family members appreciated by their Norwegian colleagues and neighbors as follows: “They like that we take care of our family” or “They usually say that we are doing good work by taking care of our parents.” Furthermore, in defense of oppressive narratives about Muslim women, a few women contended that the responsibility of being a caregiver was inherent in women instead of being due to pressure from the community.

## Discussion

This study aimed to explore the motivations and meanings of care among family caregivers of older Pakistani migrants within the Norwegian Ahmadiyya community. The conceptualization of care in the Ahmadiyya community is based on the ethics of care, which revolve around a distinctive moral framework that emphasizes the importance of caring relationships, interdependency, and concern for the care needs of others.

The participants across generations showed high care ideals (Pakistan as a reference point) and viewed caring for older family members as a familial responsibility, valuing the caregiving relationship as emphasized by the ethics of care (Noddings, 2010). Ethical relationships involve not only providing physical care but also nurturing emotional connections and understanding care recipients’ needs and preferences (Held, 2010; Tronto, 2010). The notion that children are expected to reciprocate their parents’ financial and emotional investments is not uncommon in South-Asian culture (Arora et al., 2020; Næss & Vabø, 2014). Consistent with this, reciprocal care for parents was expressed as a moral thing to do.

Studies have also shown that there exist ideas about gendered styles of caregiving (Carroll & Campbell, 2008). A systematic review study pointed out that women considered themselves quicker to react to their parents’ needs due to their emotional involvement in caregiving (Zygouri et al., 2021). In line with this, the family caregivers in our study felt that they, being women, are naturally inclined to show greater attentiveness to the care needs of their parents compared to their male siblings. However, caregiving by women was also tied to social norms of being considered a good daughter and being responsible for the transmission of moral values within the family. Such ideas were further reinforced through religious norms, highlighting the power of social norms in shaping ideas of gendered morality of care.

The family was considered a natural caregiver, where family caregivers’ position as being from a migrant background played a role in enhancing feelings of obligation and reciprocity toward their first-generation parents. Though the identity of being a migrant plays a role in conceptualizing care, it also intersects with other categories, such as gender, religion, and power structures, in shaping caregiving as a moral practice. We see that through several empirical studies among migrants in Europe, even though participants mention and emphasize “family” for caregiving, it was mainly the women who were considered caregivers (Ahmad et al., 2022; Arora et al., 2020). The idea of women being better caregivers was perceived as intrinsic and natural to women, which aligns with Gilligan’s (1982) notion of the feminine ethics of care.

Religion and repertoires are used to rationalize and cope with caregiving (Ahaddour et al., 2020; Ahmad et al., 2022). Religion was then utilized to conceptualize caregiving as a moral practice. Since most of the participants were practicing Muslims, the tenets of Islam remained a crucial aspect when making moral judgments. A study (Ismail, 2021) conducted among Arab Muslims in Denmark found that elderly care was perceived as honoring God and as a duty from God. Religion conceptualized not only caregiving as a moral practice but also defined good care, where gratitude to parents was equated with obedience without complaints while providing care (Ismail, 2021). Within the religious context, religious leaders and older community members hold the power to define what is good care and who should provide that care.

In our study, qualities such as selflessness, tolerance, patience, and sacrifice among caregivers were competencies both expected and validated by both caregivers and religious leaders as moral qualities for providing good care. It is likely that religion, on account of its normative prescriptions, further validates caregiving as a moral practice/standard and thereby makes it less likely to be challenged. Also, the caregivers perceived themselves as better suited for the caregiving role because of these qualities. Research has postulated that migrating due to religious persecution from Pakistan makes it even more critical for Ahmadis to practice their faith more vigorously now that they have gained religious freedom in the new country (Ahmed-Ghosh, 2004, 2006; Islam, 2019). It is plausible that being a “double minority” contributes to utilizing religion more heavily, including shaping ideas of good care.

However, this does not mean that the perception of caregiving as intrinsic and natural to women was shaped solely through ideas of femininity or religion, as disrupting the division and hierarchy of labor was viewed as inviting conflict in our study. This highlights the role of power differences inculcated through both gender social norms within the family and through migrant community leaders who espoused these values. Chang (1996) argued that care arises not from women’s moral concern for sustaining human connection but from the necessity for survival in oppressive relationships with men (Chang, 1996). A study conducted about Taiwanese family caregivers in the United States showed that daughters-in-law suppressed their genuine emotions of stress and anger and acted submissively in front of their in-laws for the sake of relational harmony and peaceful transnational households (Gu, 2018). This resonates with our findings about fear among some caregivers (who migrated to Norway as adults under family reunification) of avoiding their caregiving responsibilities for their mothers-in-law. Women’s lower social status and less independent access to money, power, and authority in society result in higher engagement in emotional work (Hochschild, 2012). The role of women as family caregivers may also be perpetuated by patriarchal system of oppression, rooted in cultural and religious beliefs, making these responsibilities intrinsic parts of their identity (Ahmed-Ghosh, 2004). Power was thus intersectional and facilitated the conceptualization of care as a moral practice.

The intersections of different social identities make family caregivers adhere to the prescribed standards of moral practice in the community, giving them the identity of a moral person. A study of dementia caregivers reported that family caregivers often feel morally superior as more loving and caring children compared to siblings who do not provide care (Ahmad et al., 2020). For some caregivers, moral superiority enhanced their personal growth, satisfaction, and closer relationships with care receivers on the one hand (Zarzycki et al., 2022). On the other hand, it prevents caregivers from requesting assistance from family members and professionals, resulting in a higher burden of care on them (Ahmad et al., 2020), or like in our study, they are likely to choose a less time-demanding career path with increasing care demands at home.

We also see that being a migrant, as well as being a “double minority,” resulted in more closely knit relationships. Close-knit communities can help provide familiarity with sharing cultural background, languages, and customs, support (safety) during the challenging process of adapting to a new country, and social networking, which provides valuable information, advice, and social connections (Aarset, 2020). However, this also functioned as a system of maintaining caregiving as a moral practice through fear of community members’ gossip (Gluckman, 1963). When interpersonal relationships are central, gossip acts as a mechanism that helps regulate behaviors that complement the shared moral values of the community. It often arises in response to perceived breaches of social norms or conflicts within the community, where the role of elders and community leaders is crucial (Gluckman, 1963). The conceptualization of family care as a moral practice and a fear of gossip and judgments contribute to the continuation of care in our study and nullify the practice of being vocal about caregiving challenges.

Moreover, inconsistent with the present literature (Arora et al., 2020; Bofill-Poch, 2018; Draulans & De Tavernier, 2020; Ismail, 2021; Shrestha et al., 2023), even the younger family caregivers in our study, who were active members of Ahmadiyya community, expressed traditional gender norms. It demonstrates their exposure to a complex set of expectations, cultural values, religious duties, and care norms shaped by more than one social and cultural system of reference (Levitt, 2009; Scocco, 2024). Their preference to strongly uphold traditional caregiving norms and thereby become “good caregivers” and “good Muslims” could also be due to the general lack of recognition in the wider society being a “double minority” (Galal et al., 2016). Indeed, as a close-knit community, the Ahmadis in Oslo have been found to espouse more traditional values (Appleyard, 2015). The pronounced exclusion process among migrant communities while not living up to the prevalent care norms of their country of origin further adds to the adherence to the community’s care norms (Arora et al., 2020; Tavernier & Draulans, 2018). Although the younger generation in our study believed that adult children, rather than daughters-in-law, should undertake caregiving responsibilities for their older parents, there were no significant generational differences in the motivations for caregiving.

Contrary to this, the caregiving practice was also perceived as a source of praise from the majority (ethnic Norwegian). Negative public discourses about migrants can contribute to a perceived necessity for migrants to maintain a strong moral identity and present themselves positively through maintaining a moral identity/superiority based on providing care to older family members (Islam, 2019; Morrice, 2017; Yaqin et al., 2018). Such perceptions may be more keenly felt by Ahmadis, who may not feel represented by mainstream Muslims. Studies among second-generation Ahmadi women in Germany and Switzerland (Beyeler, 2012; Kelso, 2023) mentioned that they play a unique role in acting as the face of their religious community to confront what can be a hostile public discourse, as well as a hostile intra-ethnic (religious) discourse (Islam, 2017, 2019), and strengthen their belonging to their religious group. Individuals showcase a collective responsibility to present a positive image countering the image of being oppressed by their ethnic and religious communities (Najib & Hopkins, 2019; Page & Yip, 2017). In line with this, the participants in our study expressed a denial of power imbalances in family care.

However, the intersections of religion (Islam), gender (women), and migration (migrant) in the Norwegian context make it unfavorable for caregivers to be outspoken about the challenges they face in the public sphere, as this could result in further prejudices and negative generalization of the entire migrant community. In this sense, being a “double minority” (Ahmadiyya) paved the way for the continuance and sustenance of caregiving ideals, either through fear of gossip or validation by the majority. Further, even though some participants perceived those intrinsic feminine notions of care resonated with the ethics of care, through an intersectionality analytical lens, we see how it is not just gender or culture in isolation that contributes to the conceptualization of caregiving as a moral practice. Social locations of migration and religion also direct our attention to power, which makes the moral practice of caregiving more significant in studying Ahmadi women’s situations.

### Limitations of the Study

The focus of our study is on the Ahmadi community, which, on the one hand, adds strength, as a minority within a minority community like Ahmadiyya Muslim is rarely represented within the discourse of elderly care. On the other hand, our study is not on the wider population of Pakistani origin in Norway. Further, there might also be the risk that participants in our study saw the interviews as an evaluation of them as good caregivers, often expressed as good daughters, good daughters-in-law, good Ahmadiyya, or good Muslims. Therefore, they may have stressed their compliance with these ideals while discussing caregiving in their communities.

Also, the Mosque as a place of interview (in the first phase) might have influenced participants’ narratives, potentially confirming certain care ideals. It is also possible that the Mosque being the first place of contact continued to influence participants’ narratives to some extent although second phase interviews were conducted outside the Mosque. Nevertheless, these findings again highlight the maintenance of a moral identity through caregiving and the importance for participants to be seen to espouse this identity in public. Lastly, our study did not include any male family caregivers, whose experiences might have provided additional insights into the gendered dimensions of caregiving motivations and perceived obligations.

### Future Implications

This study allows for a more nuanced understanding of how care is constructed in minority groups and recognizes the importance of considering caregiving through a morality frame. Furthermore, by highlighting the unequal distribution of caregiving responsibilities in informal care, this study can promote initiatives facilitating a more balanced sharing of caregiving duties between men and women, hence achieving gender equality in caregiving roles. The intersections of various social locations impact the ideals of caregiving in families with a migrant background, and our findings may support policymakers in creating inclusive policies, formal care, and support mechanisms that address the challenges faced by caregivers from ethnic or religious minority groups.

## Conclusion

An intersectionality perspective highlights care as a moral practice in the Ahmadiyya community, deeply rooted within gendered, familial, and religious obligations, and intensified due to the vulnerability of elderly migrant parents. The presumed moral identity of the caregiver attributed by both minority and majority communities contributes to the continuation of care within the family system. Unequal distribution of care among women is often justified, and even advocated, by the necessity of achieving and sustaining a moral identity. Future research should explore how family caregivers practice caregiving in their daily lives and the dilemmas they face in their practical lives within the context of care as a moral practice. Further, exploring how the moral construction of care influences family caregivers’ preferences and experiences with formal health and social care will be useful.

## Data Availability

The data supporting this study’s findings are available on request from the corresponding author. The data are not publicly available due to privacy or ethical concerns.
